# Socio-economic position as a moderator of cardiometabolic outcomes in patients receiving psychotropic treatment associated with weight gain: results from a prospective 12-month inception cohort study and a large population-based cohort

**DOI:** 10.1038/s41398-021-01482-9

**Published:** 2021-06-26

**Authors:** Céline Dubath, Mehdi Gholam-Rezaee, Jennifer Sjaarda, Axel Levier, Nuria Saigi-Morgui, Aurélie Delacrétaz, Anaïs Glatard, Radoslaw Panczak, Christoph U. Correll, Alessandra Solida, Kerstin Jessica Plessen, Armin von Gunten, Zoltan Kutalik, Philippe Conus, Chin B. Eap

**Affiliations:** 1 Unit of Pharmacogenetics and Clinical Psychopharmacology, Center for Psychiatric Neuroscience, Department of Psychiatry, Lausanne University Hospital, University of Lausanne, Prilly, Switzerland; 2 Center for Psychiatric Epidemiology and Psychopathology, Department of Psychiatry, Lausanne University Hospital, University of Lausanne, Prilly, Switzerland; 3 Swiss Institute of Bioinformatics, Lausanne, Switzerland; 4 Institute of Social and Preventive Medicine (ISPM), University of Bern, Bern, Switzerland; 5 Zucker School of Medicine at Hofstra/Northwell, Department of Psychiatry and Molecular Medicine, Hempstead, New York, USA; 6 The Zucker Hillside Hospital, Department of Psychiatry, Northwell Health, Glen Oaks, New York, USA; 7 Charité Universitätsmedizin, Department of Child and Adolescent Psychiatry, Berlin, Germany; 8 Service of General Psychiatry, Department of Psychiatry, Lausanne University Hospital, University of Lausanne, Prilly, Switzerland; 9 Service of Child and Adolescent Psychiatry, Department of Psychiatry, Lausanne University Hospital, University of Lausanne, Lausanne, Switzerland; 10 Service of Old Age Psychiatry, Department of Psychiatry, Lausanne University Hospital, University of Lausanne, Prilly, Switzerland; 11 University Center for Primary Care and Public Health, University of Lausanne, Lausanne, Switzerland; 12 Center for Research and Innovation in Clinical Pharmaceutical Sciences, University of Lausanne, Lausanne, Switzerland; 13 School of Pharmaceutical Sciences, University of Geneva, Geneva, Switzerland; 14 Institute of Pharmaceutical Sciences of Western Switzerland, University of Geneva, University of Lausanne, Geneva, Switzerland

**Keywords:** Schizophrenia, Neuroscience, Bipolar disorder, Depression

## Abstract

Weight gain and metabolic complications are major adverse effects of many psychotropic drugs. We aimed to understand how socio-economic status (SES), defined as the Swiss socio-economic position (SSEP), is associated with cardiometabolic parameters after initiation of psychotropic medications known to induce weight gain. Cardiometabolic parameters were collected in two Swiss cohorts following the prescription of psychotropic medications. The SSEP integrated neighborhood-based income, education, occupation, and housing condition. The results were then validated in an independent replication sample (UKBiobank), using educational attainment (EA) as a proxy for SES. Adult patients with a low SSEP had a higher risk of developing metabolic syndrome over one year versus patients with a high SSEP (Hazard ratio (95% CI) = 3.1 (1.5–6.5), *n* = 366). During the first 6 months of follow-up, a significant negative association between SSEP and body mass index (BMI), weight change, and waist circumference change was observed (25 ≤ age < 65, *n* = 526), which was particularly important in adults receiving medications with the highest risk of weight gain, with a BMI difference of 0.86 kg/m^2^ between patients with low versus high SSEP (95% CI: 0.03–1.70, *n* = 99). Eventually, a causal effect of EA on BMI was revealed using Mendelian randomization in the UKBiobank, which was notably strong in high-risk medication users (beta: −0.47 SD EA per 1 SD BMI; 95% CI: −0.46 to −0.27, *n* = 11,314). An additional aspect of personalized medicine was highlighted, suggesting the patients’ SES represents a significant risk factor. Particular attention should be paid to patients with low SES when initiating high cardiometabolic risk psychotropic medications.

## Introduction

In psychiatric populations (comprising schizophrenia, bipolar disorder, major depressive disorder, and their related spectrum disorders) life expectancy is reduced by about ≥10 years versus the general population. Approximately two-thirds of this mortality is attributed to cardiovascular diseases [1]. This increase in cardiometabolic-related health problems is multidetermined, including psychiatric illness, lifestyle, and diet behaviors, resulting in a high prevalence of obesity and other cardiometabolic risk factors [2]. In addition, many psychotropic medications (most antipsychotics, some mood stabilizers, and antidepressants) can worsen weight, body mass index (BMI), waist circumference (WC), lipid, and glucose profiles [2].

Social factors, such as low educational attainment (EA) or low income, have been associated with poor mental health outcomes and depression [3]. Some studies also linked socio-economic status (SES) with the risk of or severity of symptoms in schizophrenia and other mental disorders [4, 5]. Moreover, SES is a moderator of obesity in the general population [6]. In the area of Lausanne, Switzerland, geographic clusters of high versus low BMIs were observed, which were influenced by neighborhood-level income [7]. Similar results were reported in another Swiss study, performed on young men using conscription data [8], showing substantial spatial variations in obesity risk, which increased with lower SES. These studies suggest an influence of SES on both mental health and obesity. Nevertheless, associations between SES and cardiometabolic parameters in patients treated with psychotropic medications are under-researched.

One study found that social determinants of health were inversely associated with glycated hemoglobin in first-episode psychosis [9]. However, patients had very short prior psychotropic treatment exposure and the cross-sectional design precluded the investigation of the impact of SES factors on treatment-related metabolic health evolution. Another study conducted in treated bipolar patients revealed an inverse correlation between normal weight, overweight, obesity, or extreme obesity and income level [10]. However, this finding did not remain significant when adjusting for site of inclusion and eating disorder diagnoses. Besides, no other SES factors, such as EA, occupation, or housing condition, were characterized in this study. In a third study, using multivariable modeling, housing condition was significantly associated with weight gain during 6-month olanzapine therapy in patients with schizophrenia and bipolar disorders [11].

To better address the impact of SES, we aimed to longitudinally explore whether and how SES is associated with cardiometabolic variables in a psychiatric cohort treated with psychotropic medications, which can induce weight gain, and explore mediating effects of high-, medium- and low-risk medications. Finally, we sought to validate our epidemiological associations in the UKBiobank (UKB), a very large population-based cohort. Based on the prior literature, we hypothesized that weight gain following psychotropic medication initiation would be inversely related to SES and that this effect would be most pronounced with high cardiometabolic risk medications.

## Methods

### Psychiatric population and metabolic outcomes

A departmental guideline, adopted in 2007 in the Department of Psychiatry at Lausanne University Hospital, requires the monitoring of metabolic adverse effects when patients start a psychotropic treatment known to induce weight gain and/or worsen other metabolic parameters. In- and outpatients starting such treatments are thus followed with routine check-ups at baseline and after 1, 2, 3, 6, and 12 months. Body weight, BMI, WC, blood pressure, and plasma levels of glucose and lipids (low-density lipoprotein cholesterol (LDL), high-density lipoprotein cholesterol (HDL), total cholesterol, and triglycerides) are monitored routinely at these same time points.

Informed consent was obtained from prospectively followed patients between 4/2007 and 10/2016 (PsyMetab, *n* = 1093) as previously described [12]. Because of the non-interventional post-hoc analysis design, the requirement of informed consent was waived for patients routinely assessed as part of clinical care between 4/2007 and 12/2015 (PsyClin, *n* = 714). Data use in both cohorts was approved by the Ethics Committee of the Canton of Vaud. Patients selection is described in Supplementary Fig. 1.

Medication, diagnosis, age at medication onset, smoking status, and sex were extracted from medical files and/or specific questionnaires. Diagnostic groups were established according to ICD-10 classification, and psychotropic medications were classified, independently of daily dosage, according to their risk for inducing weight gain in three categories, i.e., low-risk (e.g., amisulpride, aripiprazole, haloperidol, lurasidone, and flupentixol); medium-risk (e.g., quetiapine, risperidone, paliperidone, lithium, mirtazapine, zuclopenthixol, and levomepromazine), and high-risk (e.g., valproate, olanzapine, and clozapine) as previously described [13].

### SES: assignment of the SSEP index

Research conducted in Switzerland aimed to determine individual’s SES based on postal address [14]. An area-based index of Swiss socio-economic position (SSEP) was developed, ranging between 0 (most disadvantaged) and 100 (most privileged). The characterization of the SSEP index by place of residency was based on 2000 census data including income, education, occupation, and housing conditions. The validity of the SSEP index was then demonstrated to be associated with all-cause and cause-specific mortality [14].

To estimate patients’ SES, postal addresses were obtained and geocoded using Google API via the ggmap R package [15]. Patients with no available personal address were excluded (Supplementary Fig. 1). Each geocoded postal address was matched with a SSEP according to the minimum distance to a reference SSEP building, with a maximum distance of 130.5 meters, as previously described [14].

### Statistical analyses

Baseline demographic variables of patients were compared with their SSEP level (below or above the median SSEP) using the *χ*^2^ test of independence for categorical variables and Student’s *t*-tests for continuous variables.

Incidence of metabolic syndrome, according to the International Diabetes Federation (IDF) definition [16], as well as the incidence of each individual component of metabolic syndrome was investigated in adult patients (25–65-years old) with low versus high SSEP during the first year of treatment. The Cox proportional hazard model was used, adjusted for confounding variables (age, sex, first available BMI, diagnosis, and treatment categories).

Linear mixed effect models were then used to assess the effect of SSEP on each cardiometabolic parameter during 1–6 months of medications treatment, adjusting for confounding variables (age, sex, baseline BMI, diagnosis, and treatment categories; see Supplementary Appendix). We used the SSEP variable once on a continuous and once on a categorical scale: (a) low SSEP (i.e., SSEP below the first quartile), (b) medium SSEP (i.e., SSEP in the second or third quartile), and (c) high SSEP (i.e., SSEP above the third quartile). Analyses were performed independently in three age groups, young patients (<25 years old), adults 25 to <65 years old, and senior patients ≥65 years old, as the meaning of the SSEP construct varies for different birth cohorts [14]. In adults, subgroup analyses were also run to differentiate the effect on BMI, weight, and WC according to initial BMI and according to treatment categories. All analyses were two-sided with alpha = 0.05. Analyses were performed using the R environment for statistical computing version 3.5.2.

### Validation in the UKB sample

We sought to validate our associations in the UKB sample and also aimed to estimate a causal effect through Mendelian randomization (MR). The details of the UKB have been described elsewhere [17]. Briefly, UKB is a prospective cohort study including more than 500,000 individuals (40–69 years) recruited from the United Kingdom during 2006–2010. We selected participants according to quality measures, ethnicity, and relatedness. We then defined a psychiatric population within the UKB based on reported psychotropic medication use to evaluate the association of EA with BMI through a cross-sectional approach. Information regarding the duration of medication treatment as well as of psychiatric diseases were unknown and the UKB sample included presumably a mix of chronic and first-episode patients. EA was used as a proxy for SES, as it is one of the four criteria in the SSEP construct. This trait was also tested for association with BMI, body weight change, and WC change in the Swiss psychiatric cohort to validate its use in the UKB. First, we examined the interaction between EA and the use of weight-inducing medications with BMI. Next, we used a two-sample MR design to estimate the causal effect of EA on BMI in both participants treated with psychotropic medications with high and low propensity to induce weight gain (i.e., category 2 and 3 in Supplementary Table 1 vs. the rest of the defined psychiatric population within the UKB). In other words, we estimated the causal effect in two distinct groups, to determine whether there was a difference in the effect of EA on BMI. MR methodology has been described in detail elsewhere [18]. Briefly, MR is a statistical method applied to large-scale genetic data, which harnesses the fact that genetic variants are inherited randomly and independently from other risk factors of diseases, to estimate the causal effect of an exposure on an outcome of interest. The random distribution of genetic variants at birth minimizes the possibility of confounding or reverse causation as explanations for the link between the exposure and outcome in the same way that the allocation of a therapy in a randomized controlled trial minimizes this possibility (see Supplementary Appendix).

## Results

### Population characteristics

From the initial cohort of 1807 patients, 966 were included in the analyses (Supplementary Fig. 1 for patient selection). Clinical and demographic parameters of the cohort are described in Table 1. The median SSEP was 61.8 (range = 29.4–86.4) and was used as a threshold to describe the cohort, stratified as low SSEP (<61.8) and high SSEP (≥61.8). The median age of the cohort was 40 years (range = 13–96 years). The proportion of smokers was higher in the low than in the high SSEP group (52% versus 46%), although the difference did not reach significance. Men represented 45.8% of the cohort and were more likely to have a low SSEP than women (49.8% of men in the low versus 41.7% in the high SSEP group, p = 0.01). In addition, compared to patients with a high SSEP, patients with a low SSEP more likely suffered from psychotic disorders (40.7% versus 31.4%) and were less diagnosed with depression (15.8% versus 21.3%). The three psychotropic medication categories were not associated with SSEP groups. Most of the cohort (60.3%) received medium cardiometabolic risk psychotropic treatment, while a minority received low-risk (19.3%) or high-risk treatment (20.5%). Based on the first available observation, patients with a low SSEP had higher BMIs than those with a high SSEP (median = 23.6 kg/m^2^ versus 22.7 kg/m^2^; *p* = 0.004), with a trend for a higher prevalence of overweight or obese patients (overweight proportion: 25.9% in the low and 22.5% in the high SSEP group and obese proportion: 14.5% versus 10.7%, *p* = 0.05). The same trend was observed regarding the proportion of central obesity (44.5% versus 38.3%, *p* = 0.08), low HDL-cholesterol (14.9% versus 9.9%, *p* = 0.06), and median diastolic blood pressure (77 mmHg versus 74 mmHg, *p* = 0.05). Finally, 42% of the total sample had total hypercholesterolemia, while median systolic blood pressure and glycaemia were in the range of normal values, with none of these variables being significantly associated with SSEP groupings.

**Table 1:** Clinical and demographic parameters of the study sample according to SSEP groups.

	*N*	Total sample	Low SSEP (29.4 ≤ SSEP < 61.8)	High SSEP (61.8 ≤ SSEP ≤ 86.4)	*p*-value^a^
Age, median (range), y	966	40 (13–96)	37 (13–93)	44 (13–96)	0.06
Men, *n* (%)	966	442 (45.8)	240 (49.8)	202 (41.7)	**0.01**
Smoking, *n* (%)	816	397 (48.7)	211 (51.7)	186 (45.6)	0.09
Main diagnosis, *n* (%)	966				**0.02**
Psychotic disorders (F20-F24;F28-F29)		348 (36)	196 (40.7)	152 (31.4)	
Schizoaffective disorders (F25)		85 (8.8)	47 (9.8)	38 (7.9)	
Bipolar disorders (F30-F31)		163 (16.9)	78 (16.2)	85 (17.6)	
Depressive disorders (F32-F33)		179 (18.5)	76 (15.8)	103 (21.3)	
Organic disorders (F00-F09; F28-F29)		28 (2.9)	12 (2.5)	16 (3.3)	
Other		163 (16.9)	73 (15.2)	90 (18.6)	
Psychotropic treatment group, *n* (%)^b^	966				0.31
Low risk of WG		186 (19.3)	100 (20.8)	86 (17.8)	
Medium risk of WG		582 (60.3)	279 (57.9)	303 (62.6)	
High risk of WG		198 (20.5)	103 (21.4)	95 (19.6)	
*Metabolic parameters at first observation*^**c**^					
BMI, median (range), kg/m^2^	966	23.2 (13.3–54.1)	23.6 (14.5–54.1)	22.7 (13.3– 53.5)	**0.004**
Overweight (25 ≥ BMI < 30 kg/m^2^), *n* (%)		234 (24.2)	125 (25.9)	109 (22.5)	0.05
Obese (BMI ≥ 30 kg/m^2^), *n* (%)		122 (12.6)	70 (14.5)	52 (10.7)	
WC, median (range), cm	819	87 (28–162)	88 (45–133)	85 (43–162)	0.09
Central obesity (WC ≥ 94 cm in male or ≥88 cm in female), *n* (%)		339 (41.4)	182 (44.5)	157 (38.3)	0.08
Hypercholesterolemia (≥5 mmol/l), *n* (%)	678	285 (42)	140 (41.2)	145 (42.9)	0.70
LDL hypercholesterolemia (≥3 mmol/l), *n* (%)	639	236 (36.9)	115 (37)	121 (36.9)	1.00
HDL hypocholesterolemia (≤1 mmol/l), *n* (%)	664	82 (12.4)	49 (14.9)	33 (9.9)	0.06
Fasting hypertriglyceridemia (≥2 mmol/l), *n* (%)	664	99 (14.9)	55 (16.6)	44 (13.2)	0.26
Systolic blood pressure, median (range), mmHg	699	120 (72–206)	120 (80–180)	120 (72–206)	0.60
Diastolic blood pressure, median (range), mmHg	699	75 (40–120)	77 (44–117)	74 (40–120)	0.05
Fasting glucose, median (range), mmol/l	492	5 (3–14.9)	5 (3–14.3)	5 (3–14.9)	0.40

Low and high SSEPs indicate SSEPs lower and higher than the median SSEP, respectively.

*BMI* body mass index, *HDL* high-density lipoprotein cholesterol, *F00-F33* ICD codes, *LDL* low-density lipoprotein cholesterol, *SSEP* Swiss socio-economic position, *WC* waist circumference, *WG* weight gain.

^a^*p*-values were calculated using Student *t*-tests for continuous variables and *χ*^2^ test of independence for categorical variables. Significant *p*-values are indicated in bold.

^b^Amisulpride, aripiprazole, haloperidol, lurasidone, and flupentixol were considered as drugs with a low propensity for WG; quetiapine, risperidone, paliperidone, lithium, mirtazapine, zuclopenthixol, and levomepromazine were classified in the group with medium propensity for WG and valproate, olanzapine and clozapine were considered as having a high propensity for WG.

^c^First observation includes observations at baseline for 86.6% of the sample, at 1 month for 9.5%, and later for 3.8% of the sample.

### Longitudinal association of SSEP and cardiometabolic parameters

**Fig. 1:**
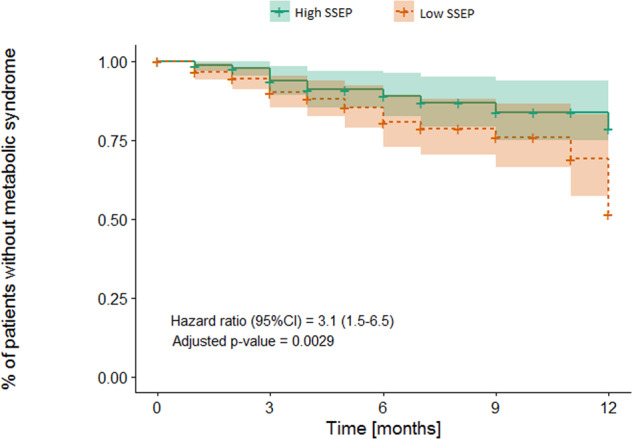
Incidence of new onset metabolic syndrome according to SSEP over one year of psychotropic treatment in the adult population. Analysis was performed in the adult population (25 ≤ age< 65) and was adjusted by age, sex, first available BMI, diagnosis, risk of psychotropic drug-induced weight gain, using a Cox proportional hazards model (*n* = 366). The number at risk at baseline: 168 High SSEP vs 198 Low SSEP, at 3 months: 98 High SSEP vs 109 Low SSEP, at 6 months: 47 High SSEP vs 54 Low SSEP, at 9 months: 29 High SSEP vs 29 Low SSEP, at 12 months: 16 High SSEP vs 16 Low SSEP. High and Low SSEP groups were defined as SSEP over (≥61.8) vs under (<61.8) median SSEP, respectively.

Although we observed significant weight gain over treatment time, no association between SSEP and cardiometabolic parameters during psychotropic treatment was observed in young patients (<25 years, *n* = 199) and senior patients (≥65 years, *n* = 204). However, significant associations were found in the adult population (*n* = 526) (Table 2). In this age group, SSEP was an important risk factor for the incidence of metabolic worsening during psychotropic treatment: among 366 adult patients without metabolic syndrome at the beginning of the psychotropic treatment, 42 new cases occurred over a one-year follow-up; patients whose SSEP was lower than the median value were three times more likely to develop metabolic syndrome compared to patients with higher SSEP (HR = 3.1, 95% CI: 1.5–6.5, Fig. 1). Results of incidence of individual risk components of metabolic syndrome are described in Supplementary Appendix and presented in Supplementary Fig. 2.

**Table 2:** Association between SSEP and BMI, weight change, and waist circumference change in the young, adult, and elderly population.

	BMI (kg/m^2^)	Weight change (%)	WC change (%)
Young (13 ≤ age<25)	*N* = 199	*N* = 199	*N*^a^ = 145
SSEP, E (95% CI)	0 (−0.015; 0.015)	0.009 (−0.057; 0.076)	0.035 (−0.254; 0.326)
Low vs medium SSEP, E (95% CI)	−0.39 (−0.84; 0.05)	−1.65 (−3.62; 0.30)	3.15 (−5.96; 12.10)
Low vs high SSEP, E (95% CI)	−0.18 (−0.68; 0.32)	−0.50 (−2.70; 1.66)	−0.88 (−10.72; 8.89)
Adult (25 ≤ age<65)	*N* = 526	*N* = 526	*N*^a^ = 390
SSEP, E (95% CI)	0.017 (0.004; 0.030)*^b^	0.063 (0.010; 0.116)*	0.141 (0.044; 0.244)**
Low vs medium SSEP, E (95% CI)	0.12 (−0.20; 0.42)	0.39 (−0.86; 1.61)	4.18 (1.87; 6.50)***
Low vs high SSEP, E (95% CI)	0.44 (0.06; 0.84)*	1.60 (0.09; 3.17)*	3.17 (0.25; 6.10)*
Senior (65 ≤ age < 97)	*N* = 204	*N* = 204	*N*^a^ = 117
SSEP, E (95% CI)	−0.001 (−0.021; 0.018)	0.011 (−0.075; 0.095)	−0.077 (−0.229; 0.083)
Low vs medium SSEP, E (95% CI)	0.31 (−0.21; 0.83)	1.23 (−1.02; 3.51)	1.35 (−2.88; 5.63)
Low vs high SSEP, E (95% CI)	−0.12 (−0.68; 0.45)	−0.36 (−2.79; 2.11)	−2.12 (−6.74; 2.43)

Weight and WC change (in %) were calculated as the difference between the current value and the baseline value divided by the baseline value.

Analyses were performed during a 6-month follow-up period, adjusted by age, sex, first available BMI, diagnosis, risk of psychotropic drug-induced weight gain and were performed using linear mixed models adjusted in a Bayesian framework and using 1,000,000 Markov chain Monte Carlo iterations. SSEP effect was estimated (E (95% CI)) on a continuous and categorical scale (three SSEP categories: first quartile defines low SSEP, second and third quartiles medium SSEP, and fourth quartile high SSEP).

*BMI* body mass index, *SSEP* Swiss socio-economic position, *WC* waist circumference.

Significant *p*-values are indicated as *p ≤ 0.05; **p ≤ 0.01; ***p ≤ 0.001.

^a^The number of patients included in this analysis was lower than for BMI and weight because of missing WC data.

^b^To understand the magnitude of these results, one can imagine a fictional patient with a baseline BMI of 20, taking a high cardiometabolic risk psychotropic treatment and with a SSEP of 30 (lowest SSEP value in the adult cohort). His/her BMI would increase by 2.2 kg/m^2^ (95% CI BMI at 6 months: 21.8–22.6) in 6 months to a value of 22.2 kg/m^2^, while the same patient with a SSEP of 86 (highest SSEP value of the adult cohort) would increase his/her BMI by 1.2 kg/m^2^ (95% CI BMI at 6 months: 20.1–22.4) in 6 months to a value of 21.2 kg/m^2^, translating into a 1 kg/m^2^ BMI difference after 6 months of treatment attributable to their SSEP difference.

Continuous SSEP were significantly negatively associated with cardiometabolic parameters in adult patients, with an increase of 0.017 kg/m^2^ in BMI (95% CI: 0.004–0.030), 0.063% in weight change (95%CI: 0.010–0.116), and 0.141% in WC change (95% CI: 0.044–0.244) per each decrease in SSEP unit (Table 2). Negative associations were also observed when comparing SSEP groups, with higher BMI in patients with low versus high SSEP (0.44 kg/m^2^ (95% CI: 0.06–0.84)), weight change (1.60% (95% CI: 0.09–3.17)), and WC change (3.17% (95% CI: 0.25–6.10)). In Supplementary Table 2, adult patients’ characteristics are presented stratified into the three SSEP groups that were used in those analyses.

Associations between SSEP and lipid parameters, glucose levels, and blood pressure in the three age groups of the cohort are described in Supplementary Appendix and shown in Supplementary Table 3.

Subgroup analyses conducted in adult patients, stratified by categories of psychotropic treatment showed a stronger association between SSEP and BMI in patients receiving high cardiometabolic risk treatments (Table 3). Indeed, a decrease from a high to low SSEP group was associated with a 0.86 kg/m^2^ higher BMI (95% CI: 0.03–1.70) in patients receiving high-risk medications, while no significant association was found in those receiving low-risk medications. The inverse association between SSEP and WC change was relatively strong in patients receiving high as well as low cardiometabolic risk psychotropic medications (0.308% of WC increase per each SSEP unit decrease in patients receiving high-risk medications (95% CI: 0.055–0.568) and 0.374% increase in patients receiving low-risk medications (95% CI: 0.074–0.675)). Analyses stratified by initial BMI are described in Supplementary Appendix and in Supplementary Table 4.

**Table 3:** Association between SSEP and BMI, weight change, and waist circumference change stratified by psychotropic medication groups in the adult population.

	BMI (kg/m^2^)	Weight change (%)	WC change (%)
*High risk drug group*^b^	*N* = 99	*N* = 99	*N*^a^ = 69
SSEP, E (95% CI)	0.026 (−0.002; 0.056)	0.069 (−0.044; 0.178)	0.308 (0.055; 0.568)*
low vs medium SSEP, E (95% CI)	0.09 (−0.60; 0.81)	0.46 (−2.28; 3.21)	5.29 (−0.66; 11.27)
low vs high SSEP, E (95% CI)	0.86 (0.03; 1.70)*	2.40 (0.91; 5.58)	6.27 (−0.99; 13.62)
*Medium risk drug group*^b^	*N* = 307	*N* = 307	*N*^a^ = 230
SSEP, E (95% CI)	0.019 (0.001; 0.037)*	0.078 (0.006; 0.151)*	0.059 (−0.042; 0.164)
low vs medium SSEP, E (95% CI)	0.19 (−0.24; 0.61)	0.77 (−1.00; 2.46)	3.24 (0.73; 5.74)*
low vs high SSEP, E (95% CI)	0.46 (−0.07; 0.97)	1.86 (−0.28; 3.98)	2.14 (−0.90; 5.19)
*Low-risk drug group*^b^	*N* = 120	*N* = 120	*N*^a^ = 91
SSEP, E (95% CI)	0.003 (−0.028; 0.034)	0.024 (−0.093; 0.139)	0.374 (0.074; 0.675)*
low vs medium SSEP, E (95% CI)	0.03 (−0.63; 0.68)	−0.05 (−2.56; 2.36)	6.21 (0.22; 12.34)*
low vs high SSEP, E (95% CI)	−0.004 (−0.905; 0.920)	0.34 (−3.09; 3.79)	5.53 (−3.34; 14.46)

Weight and WC change (in %) were calculated as the difference between the current value and the baseline value divided by the baseline value.

Analyses were performed in the adult population (25 ≤ age < 65) during a 6-month follow-up period and adjusted by age, sex, first available BMI, diagnosis and were performed using linear mixed models adjusted in a Bayesian framework and using 1,000,000 Markov chain Monte Carlo iterations. SSEP effect was estimated (E (95% CI)) on a continuous and categorical scale (three SSEP categories: first quartile defines low SSEP, second and third quartiles medium SSEP, and fourth quartile high SSEP).

*BMI* body mass index, *SSEP* Swiss socio-economic position, *WC* waist circumference, *WG* weight gain.

Significant *p*-values are indicated as *p ≤ 0.05.

^a^The number of patients included in this analysis was lower than for BMI and weight because of missing WC data.

^b^Risk of drug-induced weight gain differs among psychotropic drugs: High-risk drug group includes patients taking valproate, olanzapine, or clozapine; medium-risk drug group includes patients taking quetiapine, risperidone, paliperidone, lithium, mirtazapine, zuclopenthixol, or levomepromazine; low-risk drug group includes patients taking amisulpride, aripiprazole, haloperidol, lurasidone, or flupentixol.

The association of EA with BMI, weight change, and WC change was calculated in a small subset of PsyMetab and PsyClin participants (*n* = 199), and the results are presented in Supplementary Table 5.

### Epidemiological validation in the UKB

30,334 participants were used for the analysis, including 18,893 controls and 11,441 cases (those taking psychotropic medications with no or low effect on weight and those taking weight increasing psychotropic medications according to Supplementary Table 1: risk 1 and 2 versus 3 and 4). We identified a significant interaction between high-risk medication use and EA on BMI (*p* = 0.047). Subgroup analyses revealed that the association between EA and BMI was stronger in cases (beta: 0.10 SD BMI per 1 SD decrease in age completed education; 95% CI: 0.08–0.12; *p* < 5 × 10^−16^) than controls (beta: 0.07 SD BMI per 1 SD decrease in age completed education; 95% CI: 0.05–0.09; *p* = 2.6 × 10^−14^). In other words, every year decrease in age completed education was associated with an ~0.17 kg/m^2^ increase in BMI in cases.

### Estimating the causal effect of education on BMI using MR

The causal effect was estimated in both psychiatric high-risk and low-risk medications users within the UKB. Specifically, there were 18,755 controls and 11,314 cases (slightly different than above, as not all participants passed genetic quality control filters). In both groups, we found a significant effect of EA on BMI, (Supplementary Fig. 3) where the causal effect was stronger in cases (beta: −0.47 SD EA per 1 SD BMI; 95% CI: −0.59 to −0.34; *p* = 1.3 × 10^−13^) than in controls (beta: −0.36 SD EA per 1 SD BMI; 95% CI: −0.46 to −0.27; *p* = 8.2 × 10^−14^), consistent with the epidemiological analyses. However, the difference between the two estimates was not statistically significant (one-tailed, *t*-test *p* = 0.101).

## Discussion

This study revealed that during the first year of observed psychotropic treatment with cardiometabolic risk potential, adults aged 25–65 years with a low SSEP were three times more susceptible to developing metabolic syndrome compared to patients with high SSEP. In addition, these same patients with a low SSEP were particularly susceptible to having a higher BMI, increased body weight and increased WC when prescribed high-risk psychotropic medications. We observed consistent results in the replication analyses in the UKB.

Slightly more women were included in this study, in line with research conducted in Switzerland showing higher utilization of mental health services by women [19]. Notably, women were overrepresented in the high SSEP group. A high SES had previously been associated with better mental health outcomes and interestingly, women with schizophrenia were reported to have a better prognosis than men (with higher remission rates, fewer relapses) [3, 20, 21]. Whether women are relatively biologically protected (e.g., via estrogen), or benefit from a more favorable environment that has a positive impact on mental illness evolution, or whether having a better recovery enables them to attain (or maintain) a higher SES remains to be explored.

The median baseline BMI of the entire cohort was significantly higher in patients with a low SSEP. This result is consistent with data from the general population [6], and could be in part due to the influence of SSEP on weight gain induced by previously prescribed psychotropic medications. Indeed, most patients had already been hospitalized before study recruitment and were not medication-naïve.

No significant association between SSEP and any cardiometabolic parameters during treatment with the studied psychotropic medications was found in young and in elderly patients. However, no information was available on whether young patients were still living with their parents but, according to the Swiss Federal Statistical Office, only 20% of young people aged 18–24 years live alone in Switzerland, suggesting that a significant proportion of young patients likely still lived with their parents [22]. For such patients, the calculated SSEP actually reflected the SES of their parents and thus might differ from their own SES. Similarly, some elderly patients might also live with their children caring for them, which would also not reflect their own SES. Moreover, elderly patients living in medico-welfare establishments were excluded, as the institutional address would indeed not reflect their personal SES. Therefore, a selection bias was likely present, excluding the oldest and more severely ill patients. Taken together, these limitations might explain the lack of association found in the young and elderly subpopulations. Besides, since compared to the adult group aged 25–65 years, the sample size was <50% for the younger and elderly sample, the impact of socioeconomic factors in these age groups should be further investigated by future larger studies.

Except for systolic blood pressure, no statistically significant association between SSEP and other cardiometabolic parameters was found (glucose, lipid levels, and diastolic blood pressure) in any of the age groups. The positive association linking high SSEP to higher systolic blood pressure is surprising and must be replicated, as the opposite relationship was reported in the general population in a recent meta-analysis [23].

Psychiatric illness is associated with a risk of weight gain, and in this study this risk increases when a psychotropic medication is introduced. Indeed, the greatest association of SSEP with BMI was found in patients receiving high-risk medications, while a weaker association and no association were found in patients taking medium-risk and low-risk medications, respectively. Importantly, we also identified a causal effect of EA on BMI in the UKB psychiatric subpopulation, as shown previously in the whole UKB [24], and found, independently of the duration of treatment, a trend for a stronger effect in participants taking high-risk weight-inducing psychotropic medications compared to those not taking such medications. Socio-economic inequalities negatively impact patients, especially when they are exposed to high-risk medications, making the most disadvantaged patients more vulnerable to medication-induced cardiometabolic adverse effects. The effect of an underlying environmental factor on BMI (in our study, SSEP or EA) seems thus to be exacerbated by the presence of an additional risk factor (in our study, a high-risk medication). This observation also implies that there is a non-negligible proportion of components leading to metabolic side effects that are modifiable. Therefore, targeted interventions could improve outcomes of patients with low levels of education and/or low SES. Among other explanations, mechanistic insight for medication-induced weight gain includes changes in appetite regulation [25, 26]. Interestingly, SES influences diet quality, where people of low SES tend to follow unhealthier diets than do people of high SES. Indeed, a recent Swiss study confirmed dietary differences according to SES indicators, namely education, income, and occupation [27]. It is therefore possible that increased appetite following psychotropic medication initiation has a greater impact on weight gain in patients whose diet is less healthy. It is unknown, however, whether, following prescription of appetite-stimulating medications, strategies to prevent weight gain (e.g., controlled diet or more physical exercise) differ between patients with low compared to higher SES. Educational interventions, which promote healthy eating, should be encouraged, although the evidence to date suggests a limited effect if the price is a deterrent. Strategies involving making healthy food financially accessible, especially for patients with lower SES, may be a worthwhile endeavor [28].

The present study has several limitations and strengths. First, the SSEP score was developed from data recorded in 2000. Unfortunately, replication of the same SSEP construct with more recent data is not possible in Switzerland since the census method has changed. Nonetheless, as socio-economic factors have a long-term effect, having older data is still relevant. Assuming that individuals did not move before the study period, the SSEP reflects the socio-economic environment in which they grew up or spent their life prior to the intervention. Analyses with EA were consistent with findings using the SSEP with a significant association with WC and observed trends for associations with BMI and weight change. Future studies should try to determine which of the four criteria used in the SSEP construct most influences patients’ cardiometabolic outcomes (i.e., whether income, education, occupation, or housing conditions affect the patient outcome in different ways). Another limitation of the socio-economic variable used in this study is that the SSEP index is an ecological measure, with the possibility of high-SSEP individuals living in low-SSEP neighborhoods and vice versa. Besides, as the patients’ SES was defined based on their personal address, homeless patients, those living in residential facilities, and prisoners, representing a non-negligible part of the psychiatric population, were not included in the study (Supplementary Fig. 1). Moreover, both in the Swiss psychiatric cohort and in the UKB sample, information on illness duration and severity, prior medication, diet behaviors, alcohol consumption, and physical activity were not available, preventing us to adjust the analyses for their potential influence. Despite these limitations, the inception cohort design and longitudinal follow-up after treatment initiation enabled the prospective assessment of SSEP effects on the evolution of cardiometabolic parameters in response to the initiated psychotropic medication in a comprehensive Swiss psychiatric sample treated under real-world conditions. Moreover, the observed inverse association between SSEP and cardiometabolic worsening being strongest in the high weight-gain risk medication group strengthens the validity of our findings. Importantly, the influence of EA on BMI in subjects taking weight gain-inducing psychotropic medications was confirmed in a large population-based cohort.

In summary, in addition to other well-described clinical and environmental risk factors (e.g., young age, first psychotic episode, and low BMI), low SES was associated with an increased risk of worsening of cardiometabolic variables following the prescription of weight gain-inducing psychotropic medications. In all patients, cardiometabolic risk factors, including SES, should be assessed and carefully weighed versus the therapeutic benefits of the prescribed medications.

## Supplementary information

Online Supplement
