# Correction: Socio-economic position as a moderator of cardiometabolic outcomes in patients receiving psychotropic treatment associated with weight gain: results from a prospective 12-month inception cohort study and a large population-based cohort

**DOI:** 10.1038/s41398-021-01520-6

**Published:** 2021-10-07

**Authors:** Céline Dubath, Mehdi Gholam-Rezaee, Jennifer Sjaarda, Axel Levier, Nuria Saigi-Morgui, Aurélie Delacrétaz, Anaïs Glatard, Radoslaw Panczak, Christoph U. Correll, Alessandra Solida, Kerstin Jessica Plessen, Armin von Gunten, Zoltan Kutalik, Philippe Conus, Chin B. Eap

**Affiliations:** 1 Unit of Pharmacogenetics and Clinical Psychopharmacology, Center for Psychiatric Neuroscience, Department of Psychiatry, Lausanne University Hospital, University of Lausanne, Prilly, Switzerland; 2 Center for Psychiatric Epidemiology and Psychopathology, Department of Psychiatry, Lausanne University Hospital, University of Lausanne, Prilly, Switzerland; 3 Swiss Institute of Bioinformatics, Lausanne, Switzerland; 4 Institute of Social and Preventive Medicine (ISPM), University of Bern, Bern, Switzerland; 5 Zucker School of Medicine at Hofstra/Northwell, Department of Psychiatry and Molecular Medicine, Hempstead, New York, USA; 6 The Zucker Hillside Hospital, Department of Psychiatry, Northwell Health, Glen Oaks, New York, USA; 7 Charité Universitätsmedizin, Department of Child and Adolescent Psychiatry, Berlin, Germany; 8 Service of General Psychiatry, Department of Psychiatry, Lausanne University Hospital, University of Lausanne, Prilly, Switzerland; 9 Service of Child and Adolescent Psychiatry, Department of Psychiatry, Lausanne University Hospital, University of Lausanne, Lausanne, Switzerland; 10 Service of Old Age Psychiatry, Department of Psychiatry, Lausanne University Hospital, University of Lausanne, Prilly, Switzerland; 11 University Center for Primary Care and Public Health, University of Lausanne, Lausanne, Switzerland; 12 Center for Research and Innovation in Clinical Pharmaceutical Sciences, University of Lausanne, Lausanne, Switzerland; 13 School of Pharmaceutical Sciences, University of Geneva, Geneva, Switzerland; 14 Institute of Pharmaceutical Sciences of Western Switzerland, University of Geneva, University of Lausanne, Geneva, Switzerland

**Keywords:** Schizophrenia, Neuroscience, Bipolar disorder, Depression

Correction to: *Translational Psychiatry* 10.1038/s41398-021-01482-9, published online 26 June 2021

The original version of this article unfortunately contained a mistake. Due to a typesetting error, the following funding information was omitted: This work was supported in part by the Swiss National Research Foundation (CE and PC: 320030-120686, 324730-144064, 320030-173211 and CE, PC and KP: 320030_200602). The funding sources had no role in the writing of the manuscript or in the decision to submit it for publication. We apologize for the error. The original article has been corrected.

