# Supplementary material for: Socio-economic position as a moderator of cardiometabolic outcomes in patients receiving psychotropic treatment associated with weight gain: results from a prospective 12-month inception cohort study and a large population-based cohort
Source: Transl Psychiatry. 2021 Jun 26;11:360. doi: 10.1038/s41398-021-01482-9 (PMC8257637; doi:10.1038/s41398-021-01482-9)
Supplement: Supplementary file 1 — Online Supplement [file 41398_2021_1482_MOESM1_ESM.docx]

**Online Supplement:**

**Appendix: Supplementary Methods, Results and Discussion**

**Supplementary Table 1: List of psychotropic drugs used in the UKB and their corresponding weight-inducing risk (3 corresponds to highest risk, 2 to medium risk, 1 to low risk, and 0 to no expected risk) and frequency count.**

**Supplementary Table 2: Clinical and demographic parameters of the adult population (25≤age<65) according to low, medium and high SSEP groups**

**Supplementary Table 3: Association between SSEP and metabolic parameters in young, adult and elderly population**

**Supplementary Table 4: Association between SSEP and BMI, weight change and waist circumference change stratified by baseline BMI in the adult population**

**Supplementary Table 5: Association between educational attainment and BMI, weight change and waist circumference change in the adult population**

**Supplementary Figure 1: Flowchart of the study population**

**Supplementary Figure 2: Incidence of new onset metabolic syndrome components dysregulation according to SSEP over one year of psychotropic treatment in the adult population**

**Supplementary Figure 3:** **Scatter plot illustrates the MR results estimating the causal effect of educational attainment on BMI in both high risk psychotropic weight-inducing drug-users and non users**.

**Appendix**

**METHODS**

**Linear mixed effect models**

Model adjustment was carried out in a Bayesian framework and using a large number (1 million) of Markov chain Monte Carlo iterations ^1^. All Bayesian models were adjusted using the MCMCglmm package in R. Reported estimates are mode of posterior probability for each parameter accompanied with the corresponding 95% Credible Interval (CI). We observed satisfactory convergence for all models and results were not sensitive to the number of MCMC iterations.

**UKBiobank**

Samples were genotyped on either the UK Biobank Array or the UK BiLEVE array. Phasing and imputation were performed using SHAPEIT3 and IMPUTE3, respectively, against a combined haplotype reference panel including UK10K and 1000 Genomes Phase 3. Participants which had withdrawn consent as of February 20, 2020 were removed (n=141). Analyses were filtered based on the following criteria from the “ukb_sqc_v2.txt” bulk data download file containing sample quality control metrics: “in.white.British.ancestry.subset==1”, “excess.relatives=0”, and “putative.sex.chromosome.aneuploidy=0”, and a maximum unrelated sample set was determined using kinship metrics from the “ukb1638_rel_sP.txt” file with the “ukb_gen_samples_to_remove” function from the ukbtools Rpackage^2^. Phenotype variables were processed and standardized using to the PHESANT pipeline.

**Replication of epidemiological associations in UKB**

To replicate the epidemiological association found in the PsyMetab sample, we first derived psychotropic medication use variables according to the medication and health supplements data (Data Field 20003) at study baseline. Specifically, we derived four new binary variables according to reported psychiatric medication use as outlined in Supplementary Table 1 corresponding to their risk for inducing weight gain (high, medium, low and no risk), using the same approach as in PsyMetab, plus an additional category for participants taking psychotropic medications with no reported risk for inducing weight gain. Participants could be defined as “medication user” for multiple categories, as each participant can list as many medications as necessary. Next, we restricted the sample to only participants who were taking at least one of the listed medications to establish a psychiatric population within the UKB. There was no available information on the duration of treatment and the sample was most probably a mix of chronic and first episode patients. Using this subset, we then sought to evaluate the interaction effect between weight gain-inducing psychotropic medications and education on BMI in a cross-sectional analysis. High-risk medication users were defined as participants taking at least one high- or medium-risk drug, while the remaining participants were considered as low risk users (category 2 and 3 versus 1 and 2 in Supplementary Table 1). Age at completed full-time education (Data Field 845, older age indicated greater education) was used as a proxy for educational attainment (EA), and BMI was evaluated using Data Field 21001. The model was adjusted for age, sex and age squared. When a significant interaction was identified, the effect of age at completed full-time education on BMI was estimated in the two subgroups. BMI and age at completed education were standardized to have a mean of zero and a standard deviation of 1 in each subgroup.

**Mendelian randomization**

The effect of genetic variants on EA (i.e. the exposure in the MR) was obtained from the Social Sciences Genetic Association Consortium (SSGAC, <https://www.thessgac.org/data>). Specifically, we used the data for EA reported by Lee et al. for 10K SNPs (file “GWAS_EA.to10K.txt”), which included all lead SNPs (p < 5 x 10^-8^) for EA, among other inclusion criteria^3^. EA was defined as number of years of schooling completed, measured in over 1.1 million individuals. A set of instrumental variables (IVs) to be used in the MR was selected according to the following procedure. First, the dataset from SSGAC was filtered to only GW-significant SNPs (p < 5 x 10^-8^) resulting in 2415 SNPs. Second, 373 SNPs were removed because they were both palindromic (i.e. A/T or C/G SNPs) and had MAF < 0.35. SNPs matching these criteria are difficult to harmonize with external datasets, as the strand cannot be easily determined. Third, an additional 9 SNPs were removed because they were not present in the UKB v2 bgen files. Finally, SNPs in both the UKB and passing GW-significance filter were pruned for linkage disequilibrium with a window size of 10000 and r^2^ value < 0.001 according to the European samples from the 1000 Genomes Project resulting in 393 SNPs.

The effect of these 393 SNPs on BMI (i.e. the outcome in the MR) were estimated in the UKB in the two subgroups: high-risk psychiatric medication users and the rest of the psychiatric UKB cohort, as defined above. The regression models were adjusted for standard covariates, including age, age squared, sex, and the first 40 principal components, computed in each group separately. BMI was standardized to have a mean of 0 and SD of 1 in each subgroup. The effect of the SNPs on BMI was then harmonized with the EA data. Minor allele frequency (MAF) was calculated in each subgroup (users and non-users). To ensure a sufficient sample size to estimate the effect of the SNP on BMI, SNPs with MAF * n < 5 in a given subgroup were removed from the relevant MR (e.g. for a sample of 100,000, it would be necessary to retain a SNP with MAF < 5e-05). An additional 2 SNPs were removed in both subgroups, resulting in 391 SNPs to be used to estimate the causal effect of EA on BMI. Finally, MR was performed in each group using the inverse-variance weighted (fixed effects) method^4^. Pruning, harmonization and MR analysis were performed using the TwoSampleMR R-package^5^.

**Replication of results using educational attainment in PsyMetab & PsyClin**

Analyses conducted on PsyMetab and PsyClin participants using the SSEP index were replicated in a small subsample (n=199), using only EA as SES indicator. EA was defined according to the Swiss Hospital Medical Statistics ^6^ as the highest obtained degree, ranging from 1 to 6 (with 1 being no school nor vocational training completed, 2 compulsory schooling, 3 vocational training, 4 high school, 5 university of applied sciences, 6 university).

Linear mixed effect models were then used to assess the associations of EA with BMI, weight, and WC, during 1 to 6 months of treatment with the included psychotropic medications, adjusting for confounding variables (age, sex, baseline BMI, diagnosis and treatment categories) with the exact same methods as described for the analyses of the SSEP effect. We used the EA variable once on a continuous and once on a categorical scale (three EA categories: 1-2; 3-4, 5-6). Analyses were performed in adults 25 to <65 years old.

**RESULTS**

**Longitudinal association of SSEP and cardiometabolic parameters**

Incidence of dysregulation of each individual component of metabolic syndrome is presented in Supplementary Figure 2. New onset of hyperglycemia is the only component that showed a statistically significant association with SSEP, with an increased risk in patients with a lower SSEP (HR= 2·8, 95% CI: 1·01-7·5).

Besides, the linear analysis of metabolic parameters evolution showed no association with SSEP, as described in Supplementary Table 3. The only association that reached statistical significance was with systolic blood pressure in the adult cohort, where patients with a low SSEP had a 4·82 mmHg (95%CI: -0·44 to -9·22) lower systolic blood pressure compared to patients with a high SSEP, although the systolic blood pressure change from baseline to 6 months of follow-up was not associated with SSEP.

**Associations between SSEP and BMI, weight change and waist circumference change stratified by baseline BMI in the adult population**

**Subgroup analyses conducted in adult patients, stratified by initial BMI showed that SSEP was negatively associated with BMI and WC change in normal-weight patients (**0·015 kg/m^2^ BMI unit increase per each SSEP unit decrease **(95%CI: 0**·0003 to **0**·03**) and** 0·142% of WC increase per each SSEP unit decrease **(95%CI: 0**·015 to **0**·27**)), while the effect in obese patients did not reach statistical significance (Supplementary Table 4).**

**The association of EA with BMI, weight change and WC change**

Briefly, a lower EA was associated with an increase of 0·180 kg/m2 in BMI (95%CI: -0·026 to 0·382), 0·499% in weight change (95%CI: -0·321 to 1·311), and 1·658% in WC change (95%CI: 0·158 to 3·106) as shown in Supplementary table 5. The association reached statistical significance for WC change (p=0·03) with only a statistical trend observed for BMI (p = 0·08) and no significant association for weight change (p = 0·23).

**DISCUSSION**

**Longitudinal association of SSEP and cardiometabolic parameters**

The association between hyperglycemia incidence and SSEP narrowly reached statistical significance. This result is interesting and would reveal an increased susceptibility to hyperglycemia in patients with a low as compared to patients with a higher SSEP. Nevertheless, this finding was based on a small sample of participants (n=201 participants at baseline) and needs replication in a future larger study.

The absence of a statistically significant association for glucose levels and diastolic blood pressure could either be due to a too-short follow-up period (i.e, 6 months), or due to insufficient statistical power, as more data were missing for these parameters than for BMI, weight and WC. While the absence of a statistically significant association could also represent reality and highlight a specific influence of SSEP on BMI, weight and WC, these same variables are strongly associated with metabolic parameters and blood pressure, so that only other, unmeasured variables would be able to explain the dissociation between weight-based and metabolic associations with SSEP. Future studies should investigate this question in more detail.

**Associations between SSEP and BMI, weight change and waist circumference change stratified by baseline BMI in the adult population**

A low initial BMI has been consistently shown to be associated with greater psychotropic drug-induced weight gain^7, 8^, and in the present study, the observed association between SSEP and BMI was significant in normal-weight patients. In overweight and obese patients, the absence of associations between SSEP and BMI and weight gain could be due to insufficient statistical power (n=148 and n=78 overweight and obese patients, respectively, versus n=302 normal-weight patients). Alternatively, the presence of a ceiling effect would also have limited our ability to detect such associations if the psychotropic medication-related weight gain had occurred before study entry.

**REFERENCES**

1. D. Hadfield J. *MCMC methods for multi-response Generalised Linear Mixed Models: the MCM Cglmm R package*, vol. 332010.

2. Hanscombe KB, Coleman JRI, Traylor M, Lewis CM. ukbtools: An R package to manage and query UK Biobank data. *bioRxiv* 2017**:** 158113.

3. Lee JJ *et al.* Gene discovery and polygenic prediction from a genome-wide association study of educational attainment in 1.1 million individuals. *Nature genetics* 2018; **50**(8)**:** 1112-1121.

4. Burgess S, Butterworth A, Thompson SG. Mendelian randomization analysis with multiple genetic variants using summarized data. *Genetic epidemiology* 2013; **37**(7)**:** 658-665.

5. Hemani G, Tilling K, Davey Smith G. Orienting the causal relationship between imprecisely measured traits using GWAS summary data. *PLoS Genet* 2017; **13**(11)**:** e1007081.

6. Variables de la statistique médicale. Spécifications valables dès 1.1.2020. In: DFI Dfdli (ed). Office fédéral de la statistique OFS edn2020.

7. Vandenberghe F *et al.* Importance of early weight gain changes to predict long term weight gain during psychotropic drug treatment. *J Clin Psychiatry* 2015; **76**(11)**:** e1417-e1423.

8. Correll CU, Lencz T, Malhotra AK. Antipsychotic drugs and obesity. *Trends in molecular medicine* 2011; **17**(2)**:** 97-107.

**Supplementary Table 1: List of psychotropic medications used in the UKB and their corresponding weight-inducing risk (3 corresponds to highest risk, 2 to medium risk, 1 to low risk, and 0 to no expected risk) and frequency count.**

| Medication | Count | Risk | UKB coding |
| --- | --- | --- | --- |
| Valproate | 1130 | 3 | 1140872216, 1141172838, 1140872198, 1140872214, 1140872200 |
| Olanzapine | 416 | 3 | 1140928916, 1141167976 |
| Clozapine | 37 | 3 | 1141200458, 1140867420, 1140882320, 1141201792 |
| Amitriptyline | 7367 | 2 | 1140867658, 1140867934, 1140867948, 1140867938, 1140879616 |
| Mirtazapine | 1069 | 2 | 1141152732, 1141152736 |
| Lithium | 609 | 2 | 1140867490, 1140867520, 1140917270, 1140867504, 1140867518, 1140867494, 1140910976, 1140867498 |
| Clomipramine | 261 | 2 | 1140879620 |
| Quetiapine | 259 | 2 | 1141152848, 1141152860 |
| Nortriptyline | 222 | 2 | 1140867818, 1140867940, 1140867942 |
| Risperidone | 199 | 2 | 1140867444, 1141177762 |
| Imipramine | 194 | 2 | 1140879630 |
| Chlorpromazine | 139 | 2 | 1140879658, 1140910358, 1140863416 |
| Trimipramine | 71 | 2 | 1140867756, 1140867758 |
| Doxepine | 31 | 2 | 1140867640 |
| Zuclopenthixol | 26 | 2 | 1140882100, 1140867342 |
| Levomepromazine | 5 | 2 | 1140909802, 1140867122 |
| Chlorprothixene | 0 | 2 | 1140856052 |
| Flupenthixol | 80 | 1 | 1140867150, 1140867152, 1140867952 |
| Amisulprid | 68 | 1 | 1141153490, 1141184742 |
| Haloperidol | 60 | 1 | 1140867168, 1140867184, 1140867092, 1140867180 |
| Aripiprazole | 48 | 1 | 1141202024, 1141195974 |
| Sulpiride | 45 | 1 | 1140867304, 1140882376, 1140867306 |
| Promazine | 25 | 1 | 1140879746 |
| Sertindole | 0 | 1 | 1140927956, 1140927970 |
| Citalopram | 6583 | 0 | 1140921600, 1141151946 |
| Fluoxetine^1^ | 4897 | 0 | 1140879540, 1140867876, 1141174756 |
| Paroxetine | 1764 | 0 | 1140882236, 1140867888 |
| Venlafaxine | 1710 | 0 | 1140916282, 1140916288 |
| Sertraline | 1636 | 0 | 1140867878, 1140867884 |
| Diazepam | 1078 | 0 | 1140863152, 1141157496, 1140863244, 1140863250, 1140863238 |
| Escitalopram | 1022 | 0 | 1141190158, 1141180212 |
| Temazepam | 759 | 0 | 1140863202, 1140863210 |
| St. John's Wort | 663 | 0 | 1201 |
| Trazodone | 527 | 0 | 1140879634, 1140882244 |
| Duloxetine | 376 | 0 | 1141200564, 1141200570, 1141201834 |
| Nitrazepam | 225 | 0 | 1140863182, 1140863194, 1140863196 |
| Lorazepam | 125 | 0 | 1140863302, 1140863364 |
| Clobazam | 74 | 0 | 1140863268, 1140863272 |
| Oxazepam | 50 | 0 | 1140863442 |
| Reboxetine | 47 | 0 | 1141151978, 1141151982 |
| Lormetazepam | 40 | 0 | 1140863176 |
| Fluvoxamine | 27 | 0 | 1140879544, 1140867860 |
| Moclobemide | 21 | 0 | 1140867920, 1140867922 |
| Bupropion^1^ | 20 | 0 | 1141176854, 1141176858, 1141180638 |
| Flurazepam | 6 | 0 | 1140863110, 1140863112 |
| Alprazolam | 5 | 0 | 1140863308, 1140863310, 1140863238 |
| Bromazepam | 1 | 0 | 1140863318, 1140863320 |
| Clorazepate | 1 | 0 | 1140863274, 1140863276, 1140910374 |
| Ketazolam | 0 | 0 | 1140855860 |
| Prazepam | 0 | 0 | 1140855944, 1140855946 |

^1^As these drugs are known to decrease weight in some cases, a sensitivity analysis was conducted removing individuals who were taking these drugs and the interaction remained significant (n = 25,419, p = 0·020).

**Supplementary Table 2: Clinical and demographic parameters of the adult population (25≤age<65)** **according to low, medium and high SSEP groups**

|  | **N** | **Total sample** | **low SSEP^1^**  **(29.9 ≤ SSEP < 54.5)** | **medium SSEP^1^**  **(54.5 ≤ SSEP < 68.6)** | **high SSEP^1^**  **(68.6 ≤ SSEP ≤ 86.4)** | **p-value^2^** |
| --- | --- | --- | --- | --- | --- | --- |
| Age, median (range), y | 526 | 40 (25 - 64) | 36 (25 - 64) | 41 (25 - 64) | 45 (25 - 63) | **0.006**^b^ |
| Men, n(%) | 526 | 253 (48.1) | 66 (47.5) | 143 (51.1) | 44 (41.1) | 0.21 |
| Smoking, n(%) | 457 | 262 (57.3) | 73 (59.8) | 138 (58) | 51 (52.6) | 0.53 |
| Main diagnosis, n(%) | 526 |  |  |  |  | 0.59 |
| Psychotic disorders (F20-F24;F28-F29) |  | 212 (40.3) | 62 (44.6) | 110 (39.3) | 40 (37.4) |  |
| Schizoaffective disorders (F25) |  | 68 (12.9) | 20 (14.4) | 37 (13.2) | 11 (10.3) |  |
| Bipolar disorders (F30-F31) |  | 117 (22.2) | 22 (15.8) | 66 (23.6) | 29 (27.1) |  |
| Depressive disorders (F32-F33) |  | 81 (15.4) | 20 (14.4) | 43 (15.4) | 18 (16.8) |  |
| Other |  | 48 (9.1) | 15 (10.8) | 24 (8.6) | 9 (8.4) |  |
| Psychotropic medication group, n(%)^3^ | 526 |  |  |  |  | 0.10 |
| Low risk of WG |  | 120 (22.8) | 30 (21.6) | 71 (25.4) | 19 (17.8) |  |
| Medium risk of WG |  | 307 (58.4) | 75 (54) | 167 (59.6) | 65 (60.8) |  |
| High risk of WG |  | 99 (18.8) | 34 (24.5) | 42 (15) | 23 (21.5) |  |
| **Metabolic parameters at first observation^4^** |  |  |  |  |  |  |
| BMI, median (range), kg/m^2^ | 526 | 24.0 (13.6 - 53.5) | 24.0 (14.7 - 43.3) | 24.0 (13.6 - 43.7) | 23.7 (16.6 - 53.5) | 0.64 |
| Overweight (25≥BMI<30 kg/m^2^), n(%) |  | 148 (28.1) | 38 (27.3) | 84 (30) | 26 (24.3) | 0.30 |
| Obese (BMI≥30 kg/m^2^), n(%) |  | 76 (14.5) | 26 (18.7) | 38 (13.6) | 12 (11.2) |  |
| WC, median (range), cm | 462 | 88 (45 - 136) | 87 (45 - 128) | 90 (62 - 136) | 87 (62 - 121) | 0.21 |
| Central obesity (WC≥94 cm in male or ≥88 cm in female), n(%) |  | 205 (44.4) | 52 (43) | 115 (46.6) | 38 (40.4) | 0.56 |
| Hypercholesterolemia (≥5mmol/l), n(%) | 355 | 175 (49.3) | 45 (46.4) | 90 (48.7) | 40 (54.8) | 0.54 |
| LDL hypercholesterolemia (≥3mmol/l), n(%) | 334 | 148 (44.3) | 40 (43) | 77 (45.3) | 31 (43.7) | 0.93 |
| HDL hypocholesterolemia (≤1mmol/l), n(%) | 350 | 47 (13.4) | 15 (15.5) | 25 (13.9) | 7 (9.6) | 0.52 |
| Fasting hypertriglyceridemia (≥2mmol/l), n(%) | 351 | 67 (19.1) | 18 (18.8) | 41 (22.5) | 8 (11) | 0.10 |
| Systolic blood pressure, median (range), mmHg | 412 | 120 (72 - 180) | 120 (86 - 180) | 120 (80 - 174) | 116.5 (72 - 165) | 0.91 |
| Diastolic blood pressure, median (range), mmHg | 412 | 79 (46 - 120) | 80 (55 - 106) | 79 (47 - 120) | 76 (46 - 101) | 0.39 |
| Fasting glucose, median (range), mmol/l | 271 | 5 (3 - 14.9) | 5.1 (3 - 14.3) | 4.9 (3.61 - 14.9) | 4.94 (4.2 - 8) | 0.17 |
| **Metabolic parameters at last observation ^4^** |  |  |  |  |  |  |
| BMI, median (range), kg/m^2^ | 526 | 24.7 (16.2 - 45.9) | 24.6 (17.4 - 43.6) | 24.7 (16.8 - 45.9) | 24.9 (16.2 - 39.2) | 0.46 |
| Overweight (25≥BMI<30 kg/m^2^), n(%) |  | 159 (30.2) | 43 (30.9) | 84 (30) | 32 (29.9) | 1.00 |
| Obese (BMI≥30 kg/m^2^), n(%) |  | 91 (17.3) | 24 (17.3) | 48 (17.1) | 19 (17.8) |  |
| WC, median (range), cm | 470 | 91 (60 - 143) | 90 (60 - 142) | 91 (62 - 143) | 89.5 (64 - 140) | 0.77 |
| Central obesity (WC≥94 cm in male or ≥88 cm in female), n(%) |  | 243 (51.7) | 60 (50.4) | 138 (54.6) | 45 (45.9) | 0.33 |
| Hypercholesterolemia (≥5mmol/l), n(%) | 297 | 154 (51.9) | 37 (48.1) | 84 (56) | 33 (47.1) | 0.35 |
| LDL hypercholesterolemia (≥3mmol/l), n(%) | 275 | 125 (45.5) | 32 (43.8) | 68 (49.6) | 25 (38.5) | 0.31 |
| HDL hypocholesterolemia (≤1mmol/l), n(%) | 294 | 39 (13.3) | 10 (13) | 20 (13.5) | 9 (13) | 1.00 |
| Fasting hypertriglyceridemia (≥2mmol/l), n(%) | 292 | 74 (25.3) | 12 (15.6) | 44 (30.1) | 18 (26.1) | 0.06 |
| Systolic blood pressure, median (range), mmHg | 227 | 120 (82 - 180) | 116 (82 - 180) | 120 (85 - 180) | 120 (90 - 150) | 0.58 |
| Diastolic blood pressure, median (range), mmHg | 227 | 77 (46 - 120) | 76 (50 - 106) | 77.5 (46 - 120) | 78 (60 - 100) | 0.40 |
| Fasting glucose, median (range), mmol/l | 207 | 5.1 (3.1 - 9.3) | 5 (4.1 - 7) | 5.2 (3.6 - 9.3) | 5.2 (3.1 - 8.3) | 0.06 |

Abbreviations: BMI: body mass index, LDL: low-density lipoprotein cholesterol, HDL: high-density lipoprotein cholesterol, SSEP: Swiss socio-economic position, WC: waist circumference, WG: weight gain

^1^Total sample is divided into 3 groups according to the SSEP**: first quartile defines low SSEP, second and third quartiles** medium SSEP and **fourth quartile** high SSEP.

^2^p-values were calculated using ANOVA for continuous variables and χ^2^ test of independence for categorical variables. Significant p-values are indicated in bold and letters indicate which groups show significant difference calculated using Bonferroni corrected Student’s t-test: ^a^ indicates difference between low and medium SSEP, ^b^ between low and high SSEP and ^c^ between medium and high SSEP.

^3^Amisulpride, aripiprazole, haloperidol, lurasidone and flupentixol were considered as drugs with a low propensity for WG; quetiapine, risperidone, paliperidone, lithium, mirtazapine, zuclopenthixol and levomepromazine were classified in the group with moderate propensity for WG and valproate, olanzapine and clozapine were considered as having a high propensity for WG.

^4^First observation includes observations at baseline for 90% of the sample and at 1 month for 10% of the sample. Last observation includes observations up to 6 months after treatment initiation.

**Supplementary Table 3: Association between SSEP and metabolic parameters in the young, adult and elderly population**

Part A: Lipids (i.e. Total cholesterol, LDL-cholesterol, HDL-cholesterol and Triglycerides)

|  | **Total cholesterol (mmol/l)** | **LDL-cholesterol (mmol/l)** | **HDL-cholesterol (mmol/l)** | **Triglycerides (mmol/l)** |
| --- | --- | --- | --- | --- |
| **Young (**13≤age<25) | N^1^ = 149 | N^1^ = 146 | N^1^ = 148 | N^1^ = 148 |
| SSEP, E (95%CI) | 0.001(-0.012 ; 0.014) | 0(-0.011 ; 0.012) | -0.001(-0.006 ; 0.004) | 0.004(-0.006 ; 0.013) |
|  |  |  |  |  |
| low vs medium SSEP, E (95%CI) | 0.06(-0.33 ; 0.45) | 0.04(-0.31 ; 0.38) | -0.02(-0.17 ; 0.13) | 0.09(-0.20 ; 0.38) |
| low vs high SSEP, E (95%CI) | 0.19(-0.25 ; 0.61) | 0.11(-0.27 ; 0.49) | -0.02(-0.18 ; 0.16) | 0.24(-0.07 ; 0.56) |
|  |  |  |  |  |
|  | **Total cholesterol change (%)** | **LDL change (%)** | **HDL change (%)** | **Triglycerides change (%)** |
|  | N^1^ = 124 | N^1^ = 111 | N^1^ = 117 | N^1^ = 117 |
| SSEP, E (95%CI) | 0.053(-0.294 ; 0.399) | 0.305(-0.298 ; 0.898) | 0.196(-0.159 ; 0.568) | -0.254(-1.326 ; 0.814) |
|  |  |  |  |  |
| low vs medium SSEP, E (95%CI) | 0.30(-11.07 ; 11.31) | 0.04(-19.08 ; 19.53) | 3.79(-7.94 ; 15.43) | 10.02(-25.80 ; 44.81) |
| low vs high SSEP, E (95%CI) | 4.26(-7.62 ; 16.32) | 11.21(-9.36 ; 31.60) | 4.03(-8.36 ; 16.51) | 12.44(-25.35 ; 49.27) |
|  | **Total cholesterol (mmol/l)** | **LDL-cholesterol (mmol/l)** | **HDL-cholesterol (mmol/l)** | **Triglycerides (mmol/l)** |
| **Adult** (25≤age<65) | N^1^ = 400 | N^1^ = 386 | N^1^ = 401 | N^1^ = 400 |
| SSEP, E (95%CI) | -0.001(-0.011 ; 0.01) | 0.002(-0.007 ; 0.012) | -0.003(-0.007 ; 0.001) | 0.001(-0.012 ; 0.015) |
|  |  |  |  |  |
| low vs medium SSEP, E (95%CI) | -0.08(-0.33 ; 0.19) | -0.019(-0.24 ; 0.20) | -0.03(-0.11 ; 0.05) | -0.12(-0.44 ; 0.19) |
| low vs high SSEP, E (95%CI) | -0.09(-0.41 ; 0.23) | 0.02(-0.26 ; 0.30) | -0.07(-0.18 ; 0.03) | -0.02(-0.41 ; 0.38) |
|  |  |  |  |  |
|  | **Total cholesterol change (%)** | **LDL change (%)** | **HDL change (%)** | **Triglycerides change (%)** |
|  | N^1^ = 300 | N^1^ = 276 | N^1^ = 297 | N^1^ = 297 |
| SSEP, E (95%CI) | -0.023(-0.247 ; 0.201) | 0.016(-0.370 ; 0.412) | -0.063(-0.305 ; 0.172) | 0.198(-0.637 ; 1.028) |
|  |  |  |  |  |
| low vs medium SSEP, E (95%CI) | 0.75(-4.69 ; 6.08) | -0.87(-10.16 ; 8.62) | 1.02(-4.64 ; 6.77) | 3.89(-16.27 ; 23.85) |
| low vs high SSEP, E (95%CI) | 1.87(-4.84 ; 8.57) | 3.09(-8.59 ; 14.76) | 1.70(-5.24 ; 8.92) | 4.40(-19.84 ; 29.41) |
|  | **Total cholesterol (mmol/l)** | **LDL-cholesterol (mmol/l)** | **HDL-cholesterol (mmol(l)** | **Triglycerides (mmol/l)** |
| **Senior (**65≤age<97) | N^1^ = 139 | N^1^ = 134 | N^1^ = 138 | N^1^ = 136 |
| SSEP, E (95%CI) | -0.012(-0.031 ; 0.007) | -0.013(-0.029 ; 0.003) | 0(-0.007 ; 0.006) | -0.003(-0.011 ; 0.005) |
|  |  |  |  |  |
| low vs medium SSEP, E (95%CI) | 0.01(-0.52 ; 0.53) | -0.05(-0.51 ; 0.40) | -0.03(-0.21 ; 0.15) | -0.05(-0.29 ; 0.20) |
| low vs high SSEP, E (95%CI) | -0.38(-0.91 ; 0.16) | -0.44(-0.90 ; 0.03) | -0.01(-0.19 ; 0.17) | -0.15(-0.40 ; 0.09) |
|  |  |  |  |  |
|  | **Total cholesterol change (%)** | **LDL change (%)** | **HDL change (%)** | **Triglycerides change (%)** |
|  | N^1^ = 166 | N^1^ = 152 | N^1^ = 160 | N^1^ = 161 |
| SSEP, E (95%CI) | -0.167(-0.479 ; 0.142) | -0.465(-1.018 ; 0.103) | 0.140(-0.247 ; 0.537) | -0.241(-0.897 ; 0.419) |
|  |  |  |  |  |
| low vs medium SSEP, E (95%CI) | -1.26(-9.84 ; 7.61) | -2.48(-18.43 ; 13.36) | -2.76(-13.60 ; 7.75) | -4.34(-23.31 ; 14.32) |
| low vs high SSEP, E (95%CI) | -0.42(-9.10 ; 8.45) | -9.79(-26.09 ; 6.14) | 7.63(-3.12 ; 18.48) | -6.66(-25.33 ; 12.38) |

Part B: Systolic and Diastolic Blood Pressure and Glucose

|  | **Systolic blood pressure (mmHg)** | **Diastolic blood pressure (mmHg)** | **Glucose (mmol/l)** |
| --- | --- | --- | --- |
| **Young (**13≤age<25) | N^1^ = 126 | N^1^ = 126 | N^1^ = 101 |
| SSEP, E (95%CI) | -0.002(-0.180 ; 0.173) | -0.051(-0.214 ; 0.113) | 0.001(-0.008 ; 0.009) |
|  |  |  |  |
| low vs medium SSEP, E (95%CI) | 3.18(-1.87 ; 8.24) | -0.06(-4.81 ; 4.63) | 0.15(-0.12 ; 0.41) |
| low vs high SSEP, E (95%CI) | 1.05(-4.69 ; 6.88) | -0.13(-5.52 ; 5.24) | 0.11(-0.18 ; 0.40) |
|  |  |  |  |
|  | **Systolic blood pressure change (%)** | **Diastolic blood pressure change (%)** | **Glucose change (%)** |
|  | N^1^ = 113 | N^1^ = 113 | N^1^ = 72 |
| SSEP, E (95%CI) | 0.045(-0.190 ; 0.284) | -0.001(-0.349 ; 0.350) | 0.092(-0.145 ; 0.327) |
|  |  |  |  |
| low vs medium SSEP, E (95%CI) | 3.89(-2.98 ; 10.81) | -5.34(-15.46 ; 4.70) | 5(-2.42 ; 12.44) |
| low vs high SSEP, E (95%CI) | 3.19(-4.77 ; 10.94) | -0.01(-11.47 ; 11.57) | 4.27(-3.67 ; 11.89) |
|  | **Systolic blood pressure (mmHg)** | **Diastolic blood pressure (mmHg)** | **Glucose (mmol/l)** |
| **Adult** (25≤age<65) | N^1^ = 293 | N^1^ = 293 | N^1^ = 291 |
| SSEP, E (95%CI) | -0.153(-0.305 ; -0.004)* | -0.015(-0.138 ; 0.107) | 0.004(-0.006 ; 0.014) |
|  |  |  |  |
| low vs medium SSEP, E (95%CI) | -3.39(-7.04 ; 0.15) | -1.35(-4.35; 1.53) | -0.03(-0.27 ; 0.21) |
| low vs high SSEP, E (95%CI) | -4.82(-9.22 ; -0.44)* | 0.14(-3.45 ; 3.73) | 0.11(-0.19 ; 0.40) |
|  |  |  |  |
|  | **Systolic blood pressure change (%)** | **Diastolic blood pressure change (%)** | **Glucose change (%)** |
|  | N^1^ = 224 | N^1^ = 224 | N^1^ = 169 |
| SSEP, E (95%CI) | -0.051(-0.240 ; 0.147) | -0.050(-0.272 ; 0.171) | 0.025(-0.240 ; 0.280) |
|  |  |  |  |
| low vs medium SSEP, E (95%CI) | -3.21(-7.99 ; 1.42) | -3.25(-8.49 ; 2.29) | -3.99(-10.42 ; 2.47) |
| low vs high SSEP, E (95%CI) | -2.36(-8.02 ; 3.28) | -2.48(-8.95 ; 4.02) | 3.32(-4.29 ; 10.76) |
|  | **Systolic blood pressure (mmHg)** | **Diastolic blood pressure (mmHg)** | **Glucose (mmol/l)** |
| **Senior (**65≤age<97) | N^1^ = 47 | N^1^ = 47 | N^1^ = 62 |
| SSEP, E (95%CI) | -0.320(-0.882 ; 0.231) | -0.012(-0.418 ; 0.382) | -0.014(-0.037 ; 0.008) |
|  |  |  |  |
| low vs medium SSEP, E (95%CI) | -5.39(-22.15 ; 11.12) | -7.57(-18.62 ; 3.76) | 0.04(-0.58 ; 0.66) |
| low vs high SSEP, E (95%CI) | -4.58(-20.33 ; 11.16) | 3.33(-7.36 ; 13.85) | -0.18(-0.74 ; 0.39) |
|  |  |  |  |
|  | **Systolic blood pressure change (%)** | **Diastolic blood pressure change (%)** | **Glucose change (%)** |
|  | N^1^ = 32 | N^1^ = 32 | N^1^ = 29 |
| SSEP, E (95%CI) | -0.290(-1.210 ; 0.616) | -0.425(-1.417 ; 0.558) | -0.455(-1.026 ; 0.112) |
|  |  |  |  |
| low vs medium SSEP, E (95%CI) | 5.77(-26.52 ; 37.11) | 3.72(-31.03 ; 39.30) | -1.40(-20.58 ; 18.01) |
| low vs high SSEP, E (95%CI) | -10.64(-40.96 ; 18.91) | -8.68(-40.94 ; 25.11) | -4.69(-19.91 ; 10.41) |

Abbreviations: HDL: high-density lipoprotein; LDL: low-density lipoprotein; SSEP: Swiss socio-economic position

Metabolic parameter changes (in %) were calculated as the difference between the current value and the baseline value divided by the baseline value.

Analyses were performed during a 6-month follow-up period and adjusted by age, sex, first available BMI, diagnosis, risk of psychotropic drug-induced weight gain and were performed using linear mixed models adjusted in a Bayesian framework and using 1,000,000 Markov chain Monte Carlo iterations. SSEP effect was estimated (E (95%CI)) on a continuous and categorical scale (three SSEP categories: first quartile defines low SSEP, second and third quartiles medium SSEP and fourth quartile high SSEP). Significant p-values are indicated as *p≤0.05; **p≤0.01; ***p≤0.001.

^1^The number of patients included in the analyses differs because of missing data.

**Supplementary Table 4: Association between SSEP and BMI, weight change and waist circumference change stratified by baseline BMI in the adult population**

|  | **BMI (kg/m^2^)** | **Weight change (%)** | **WC change (%)** |
| --- | --- | --- | --- |
| **Obese (BMI>30 kg/m^2^)** | N = 76 | N = 76 | N^1^ = 54 |
| SSEP, E (95%CI) | 0.048(-0.011 ; 0.108) | 0.144(-0.020 ; 0.312) | 0.015(-0.177 ; 0.210) |
|  |  |  |  |
| low vs medium SSEP, E (95%CI) | -0.65(-1.92 ; 0.55) | -1.49(-4.98 ; 2.02) | 0.12(-3.72 ; 4.17) |
| low vs high SSEP, E (95%CI) | 1.34(-0.20 ; 2.89) | 3.71(-0.73 ; 8.05) | -0.76(-6.08 ; 4.77) |
| **Overweight (25≤ BMI kg/m^2^ <30)** | N = 148 | N = 148 | N^1^ = 107 |
| SSEP, E (95%CI) | 0.006(-0.018 ; 0.030) | 0.020(-0.069 ; 0.108) | 0.183(-0.043 ; 0.419) |
|  |  |  |  |
| low vs medium SSEP, E (95%CI) | 0.29(-0.24 ; 0.83) | 1.07(-0.91 ; 3.05) | 5.28(0.40 ; 10.10)* |
| low vs high SSEP, E (95%CI) | 0.20(-0.51 ; 0.91) | 0.70(-1.98 ; 3.33) | 1.50(-5.47 ; 8.66) |
| **Normal weight (BMI<25 kg/m^2^)** | N = 302 | N = 302 | N^1^ = 229 |
| SSEP, E (95%CI) | 0.015(0.0003 ; 0.030)* | 0.064(-0.007 ; 0.135) | 0.142(0.015 ; 0.270)* |
|  |  |  |  |
| low vs medium SSEP, E (95%CI) | 0.16(-0.20 ; 0.54) | 0.50(-1.18 ; 2.27) | 4.57(1.32 ; 7.76)** |
| low vs high SSEP, E (95%CI) | 0.38(-0.07 ; 0.82) | 1.57(-0.55 ; 3.61) | 4.14(0.42 ; 7.94)* |

Abbreviations: BMI: body mass index, SSEP: Swiss socio-economic position, WC: waist circumference, WG: weight gain

Weight and WC change (in %) were calculated as the difference between the current value and the baseline value divided by the baseline value.

Analyses were performed in the adult population (25≤age<65) during a 6-month follow-up period and adjusted by age, sex, first available BMI, diagnosis, risk of psychotropic drug-induced weight gain and were performed using linear mixed models adjusted in a Bayesian framework and using 1,000,000 Markov chain Monte Carlo iterations. SSEP effect was estimated (E (95%CI)) on a continuous and categorical scale (three SSEP categories: first quartile defines low SSEP, second and third quartiles medium SSEP and fourth quartile high SSEP). Significant p-values are indicated as *p≤0.05; **p≤0.01; ***p≤0.001.

^1^The number of patients included in this analysis was lower than for BMI and weight because of missing WC data.

**Supplementary Table 5: Association between educational attainment and BMI, weight change and waist circumference change in the adult population**

|  | **BMI (kg/m^2^)** | **Weight change (%)** | **WC change (%)** |
| --- | --- | --- | --- |
| **Adult** (25≤age<65) | N = 119 | N = 119 | N^1^ = 96 |
| EA, E (95%CI) | 0.180(-0.026 ; 0.382) | 0.499(-0.321 ; 1.311) | 1.658(0.158 ; 3.106)* |
|  |  |  |  |
| low vs medium EA, E (95%CI) | -0.10(-0.74 ; 0.57) | -0.39(-3.04 ; 2.17) | -0.10(-4.90 ; 4.80) |
| low vs high EA, E (95%CI) | 0.47(-0.32 ; 1.26) | 1.09(-2.13 ; 4.21) | 5.82(0.03 ; 11.79) |

Abbreviations: BMI: body mass index, EA: educational attainment, SSEP: Swiss socio-economic position, WC: waist circumference

Weight and WC change (in %) were calculated as the difference between the current value and the baseline value divided by the baseline value.

Analyses were performed during a 6-month follow-up period, adjusted by age, sex, first available BMI, diagnosis, risk of psychotropic drug-induced weight gain and were performed using linear mixed models adjusted in a Bayesian framework and using 1,000,000 Markov chain Monte Carlo iterations. Educational attainment effect was estimated (E (95%CI)) on a continuous and categorical scale (three EDU categories: 1-2 defines low EDU, 3-4 medium EDU and 5-6 high EDU). Significant p-values are indicated as *p≤0.05.

^1^The number of patients included in this analysis was lower than for BMI and weight because of missing WC data.


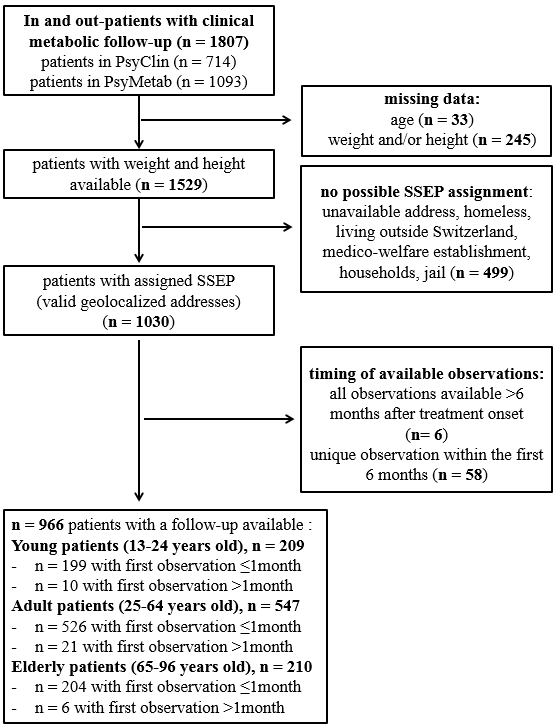
**Supplementary Figure 1:** Flowchart of the study population


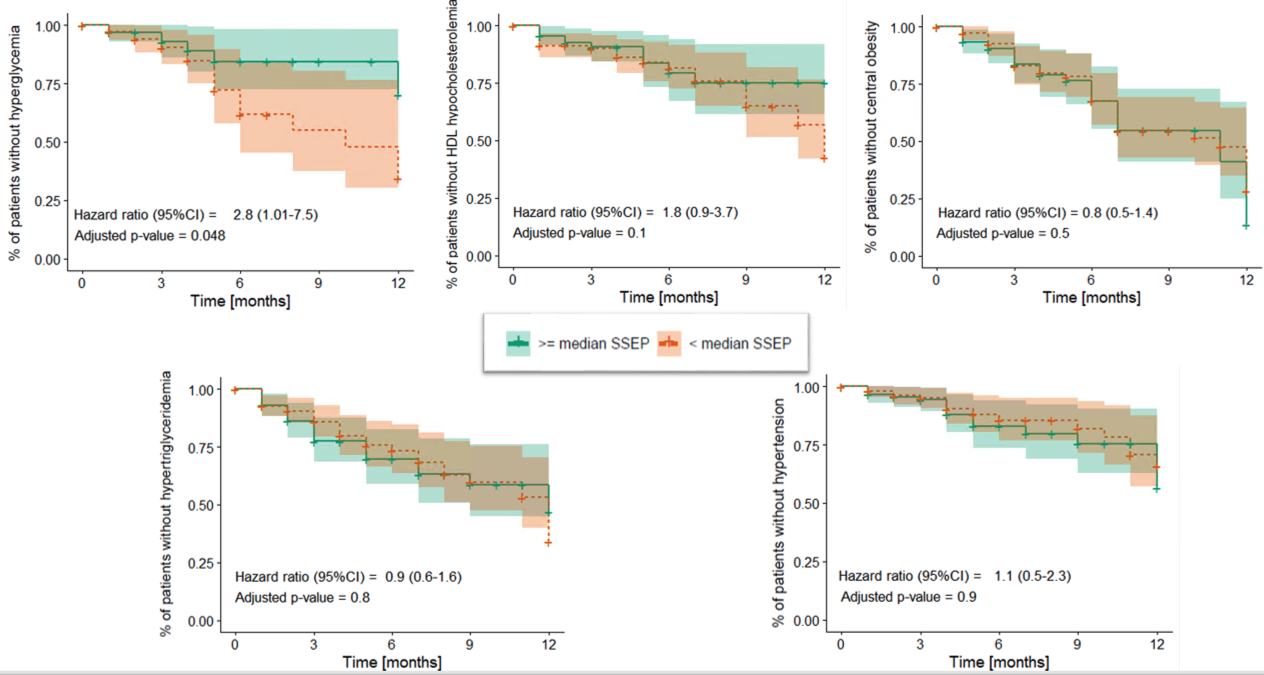
**Supplementary Figure 2: Incidence of new onset metabolic syndrome components dysregulation according to SSEP over one year of psychotropic treatment in the adult population**

Analysis was performed in the adult population (25≤age<65) and was adjusted by age, sex, first available BMI, diagnosis, risk of psychotropic drug-induced weight gain, using a Cox proportional hazards model. Number at risk for each analysis: **Hyperglycemia**: at baseline: 95 High SSEP vs 106 Low SSEP, at 3 months: 44 High SSEP vs 50 Low SSEP, at 6 months: 14 High SSEP vs 13 Low SSEP, at 9 months: 9 High SSEP vs 8 Low SSEP, at 12 months: 6 High SSEP vs 7 Low SSEP; **HDL hypocholesterolemia**: at baseline: 100 High SSEP vs 121 Low SSEP, at 3 months: 52 High SSEP vs 70 Low SSEP, at 6 months: 20 High SSEP vs 34 Low SSEP, at 9 months: 12 High SSEP vs 21 Low SSEP, at 12 months: 9 High SSEP vs 12 Low SSEP; **Central obesity**: at baseline: 94 High SSEP vs 113 Low SSEP, at 3 months: 53 High SSEP vs 67 Low SSEP, at 6 months: 26 High SSEP vs 44 Low SSEP, at 9 months: 12 High SSEP vs 19 Low SSEP, at 12 months: 6 High SSEP vs 10 Low SSEP; **Hypertriglyceridemia**: at baseline: 108 High SSEP vs 131 Low SSEP, at 3 months: 60 High SSEP vs 77 Low SSEP, at 6 months: 23 High SSEP vs 34 Low SSEP, at 9 months: 14 High SSEP vs 20 Low SSEP, at 12 months: 10 High SSEP vs 14 Low SSEP; **Hypertension**: at baseline: 101 High SSEP vs 129 Low SSEP, at 3 months: 46 High SSEP vs 59 Low SSEP, at 6 months: 16 High SSEP vs 22 Low SSEP, at 9 months: 13 High SSEP vs 17 Low SSEP, at 12 months: 9 High SSEP vs 14 Low SSEP. High and Low SSEP groups were defined as SSEP over (≥61.8) vs under (<61.8) median SSEP, respectively.

**Supplementary Figure 3**: Scatter plot illustrates the MR results estimating the causal effect of educational attainment on BMI in both high risk psychotropic weight-inducing drug-users and non users. Each point represents a SNP, where the x-axis illustrates the effect of the SNP on EA as calculated in SSGAC and the y-axis illustrated the effect on BMI in the UKB. The regression line represents the overall causal effect in each subgroup. We observed a stronger effect in high risk drug-users as compared with non-high risk drug users, as seen by the difference between the two slopes of the lines.
